# Significance of radiofrequency ablation in large solid benign thyroid nodules

**DOI:** 10.3389/fendo.2022.902484

**Published:** 2022-10-17

**Authors:** Yan Lin, Yao-ping Shi, Xiao-yin Tang, Min Ding, Yi He, Ping Li, Bo Zhai

**Affiliations:** Department of Interventional Oncology, Renji Hospital, Shanghai Jiao Tong University School of Medicine, Shanghai, China

**Keywords:** large benign solid thyroid nodules, radiofrequency ablation, volume reduction rate, thyroid function, RFA

## Abstract

**Objective:**

The aim of this study is to explore efficacy and safety for radiofrequency ablation (RFA) among cases attacked by large benign solid thyroid nodules, mainly focusing on volume reduction, complication rate, and thyroid function.

**Methods and materials:**

From June 2015 to November 2019, 24 patients with 25 large benign solid thyroid nodules (more than 25 ml) underwent single or sequential RFA in our institution. Eleven nodules achieved complete ablation after single RFA, whereas the other 14 nodules received sequential RFA. Volume reduction in large nodules was evaluated. Following single or sequential RFA, all patients received clinical and ultrasound evaluations, and the median follow-up duration among them was 23.5 months. Technical success, complication rate, and recurrence rate were assessed as well.

**Results:**

In single RFA group, volume reduction ranged from 62.6% to 99.4% (mean ± SD, 93.6 ± 9.9%) 6 months after RFA. In sequential RFA group, volume reduction ranged from 30.6% to 92.9% (mean ± SD, 67.4 ± 17.8%) after the first RFA and was between 83.4% and 98.4% (mean ± SD, 94.8± 3.8%) 6 months after the second RFA. The concentrations of FT3 and FT4 increased slightly 1 day after RFA and returned to normal level 1 month after.

**Conclusions:**

Single or sequential RFA is safe and effective in treating large benign solid thyroid nodules (more than 25 ml) that cause obvious compressive symptoms. Hence, compression symptoms and cosmetic conditions could be effectively improved through single or sequential RFA without marginal recurrence.

## Introduction

Thyroid nodule is a common pathological condition, and detection rate for this condition is increased with advancements in medical imaging technologies. The prevalence of thyroid nodule is reportedly up to 40%–50% (1–3). Most benign nodules show no symptoms and need merely routine monitoring. However, around one-fifth of thyroid nodules would expand (4). The growth of thyroid nodules may lead to neck discomfort and cosmetic complaints, affecting patients’ daily life. Thyroid nodules near airway outside thorax would squeeze upper respiratory tract and cause breathing problems to different degrees (5). Therefore, large symptomatic benign thyroid nodules should be treated actively and timely (6, 7).

Until now, total or partial thyroid surgery has become the predominant therapeutic method for symptomatic thyroid nodules, and surgery would be conducted merely when nodules grow large (with a diameter more than 4 cm) or cause obstructive and/or squeezing symptoms (4, 8, 9). Conventional surgical operation could delete symptomatic nodules and decrease malignant risks, but complications (impermanent and constant) would attack 2%–10% of the patients, including scar formation and iatrogenic hypothyroidism (10–12). Iatrogenic hypothyroidism represents one ineluctable phenomenon following total thyroidectomy and needs to be treated with lifelong L-thyroxine replacement. Because of the risk of complications, surgery is not proper among high-risk cases and those worrying about operative complications.

In recent years, with the development of interventional ultrasound, minimally invasive measures, like ethanol ablation, laser ablation, and radiofrequency ablation (RFA), are applied in treating thyroid nodules (13–17). RFA could be applied for many kinds of benign lesions and malignancies (18–22). Because of its advanced efficacy and safety and satisfactory cosmetic outcome without incisions, RFA is also widely used to treat benign thyroid nodules and malignant thyroid nodules unsuitable for surgical operations and has achieved a robust improvement in handling nodule-associated compression and cosmetic issues through decreasing nodule volumes (23–25). Single RFA is efficient for small- or medium-sized thyroid nodules, and volume reduction ratio of this measure is estimated to reach 84.1% and 79.4%, according to South Korean (23) and Italian teams (25), separately. However, large thyroid nodules may require multiple treatment sessions (24), more safety concerns, and higher ablation techniques.

**Figure 1:**
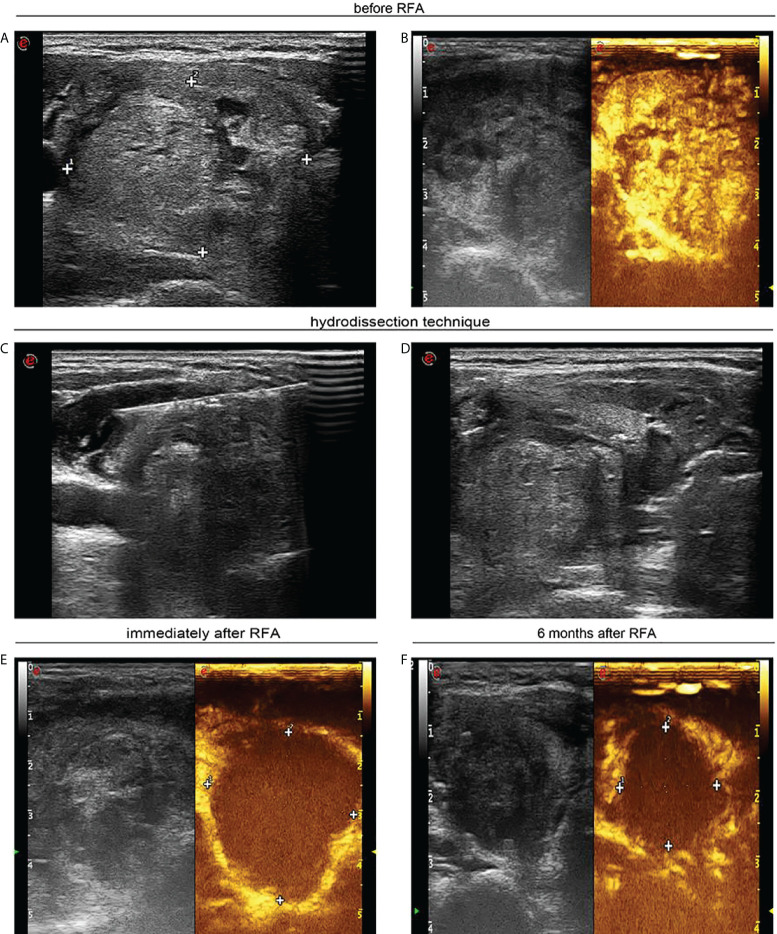
A 40-year-old woman with a large solid thyroid nodule received ultrasound-guided radiofrequency ablation. **(A)** Ultrasound image in transverse view unveils a solid thyroid nodule of 4.0 × 3.4 cm prior to radiofrequency ablation. **(B)** Contrast-enhanced ultrasound shows heterogeneous hyperenhancement in large thyroid nodule. **(C)** Ultrasound image in transverse view displays that sterile water was injected between thyroid tissue and carotid artery. **(D)** Ultrasound image in transverse view confirms that sterile water has reached between thyroid tissue and trachea. **(E)** Contrast-enhanced ultrasound indicates no enhancement in ablation zone following radiofrequency ablation. **(F)** Ultrasound image in transverse view indicates that the nodule was decreased to 2.1 × 1.8 cm 6 months after percutaneous radiofrequency ablation.

**Figure 2:**
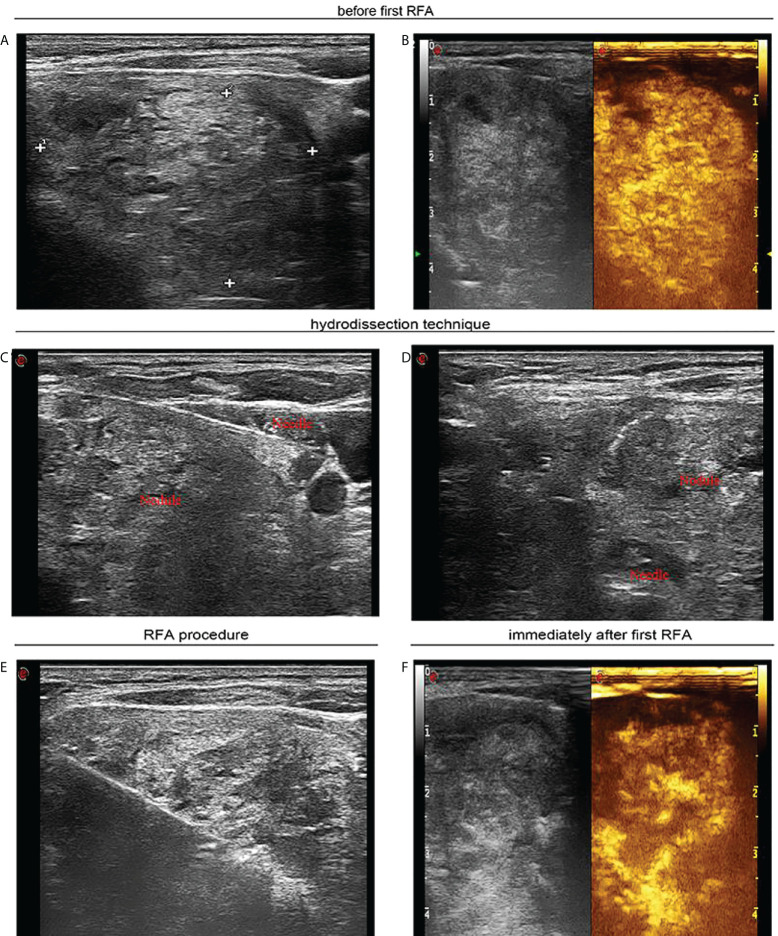
A 55-year-old woman with a large solid thyroid nodule extending to subclavian region received ultrasound-guided sequential radiofrequency ablation treatment. **(A)** Before the first radiofrequency ablation ultrasound image in transverse view unveils a solid thyroid nodule of 4.2 × 3.9 cm that extended to the subclavian region. **(B)** Contrast-enhanced ultrasound manifests hyperenhancement in large thyroid nodule. **(C)** Ultrasound image in transverse view confirms that sterile water has reached between thyroid tissue and carotid artery. **(D)** Ultrasound image in transverse view reveals that sterile water has reached between thyroid tissue and trachea. **(E)** Ultrasound image in transverse view shows that percutaneous radiofrequency ablation was implemented utilizing the moving shot technique. **(F)** Contrast-enhanced ultrasound indicates residual enhancement in partial thyroid nodule extending below clavicle immediately after the first radiofrequency ablation.

**Figure 3:**
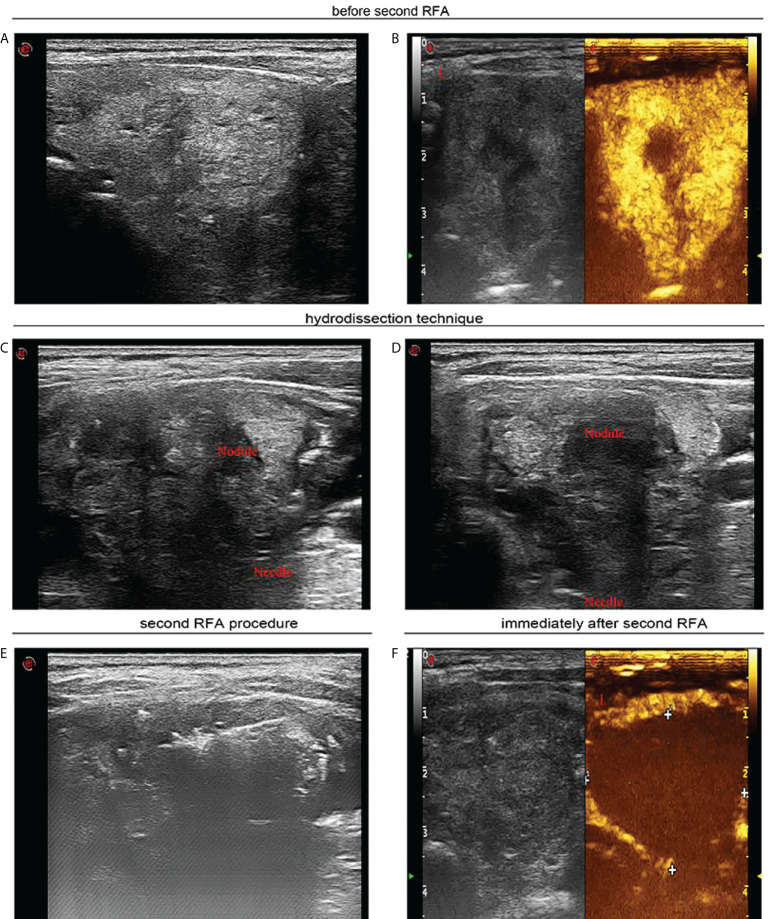
Seven months after first radiofrequency ablation, a 55-year-old woman received sequential radiofrequency ablation treatment. **(A)** Before second radiofrequency ablation ultrasound image in transverse view uncovers a heterogeneous hyperechoic nodule thyroid nodule of 3.5 × 2.6 cm. **(B)** Contrast-enhanced ultrasound indicates hyperenhancement in peripheral area of the thyroid nodule, and there was no enhancement in the central area of the nodule. **(C)** Ultrasound image in transverse view indicates that sterile water has reached between thyroid tissue and carotid artery. **(D)** Ultrasound image in transverse view confirmed that sterile water has reached between thyroid tissue and trachea. **(E)** Ultrasound image in transverse view demonstrates that percutaneous radiofrequency ablation was implemented through moving shot technique. **(F)** Contrast-enhanced ultrasound shows that immediately after the second radiofrequency ablation, there was no enhancement in the part of the thyroid nodule extending below the clavicle.

In our research, we retrospectively checked efficacy and safety for single or sequential RFA treatment regimen to handle large benign solid thyroid nodules with a volume more than 25 ml. Adopting single RFA, 11 large thyroid nodules in 11 patients were completely ablated, but 14 large nodules adjacent to the trachea or extending to subclavian in 13 patients were not completely destroyed for security reasons. Before sequential RFA, these patients received ultrasound US examination to verify that the absorption of ablation area met safety requirements for the second RFA (**Figure 1–3**). In both groups, all large benign solid thyroid nodules were destroyed completely, and nodule volume was fully compressed without significant influence on thyroid function. Therefore, assisted with multiplanar hydrodissection technique, RFA regimen is safe and efficient for large benign solid thyroid nodules.

## Materials and methods

Our retrospective research obtained permission from the Institutional Review Board of Renji Hospital. From June 2015 to November 2019, 24 patients (six men and 18 women; aged between 23 and 85 years; average age, 52.4 years) having 25 large benign solid thyroid nodules received single or sequential RFA treatment in our department. The inclusion and exclusion criteria for enrolled cases were summarized in **Table 1**.

**Table 1:** The inclusion and exclusion criteria for the included patients.

Inclusion criteria	Exclusion criteria
(1) Large solid thyroid nodules with the volume more than 25 ml; (2) benign lesions confirmed by US examinations; (3) reaching normal and clear cytologic results in US-guided core-needle biopsy; (4) ordinary serum thyroid hormone degree and normal calcitonin level; and (5) obvious neck symptoms or cosmetic concerns.	(1) Contraindications to RFA; (2) serious cardiopulmonary diseases; (3) severe coagulation disorders; (4) without available clinical records; and (5) unwilling to receive RFA.

In this retrospective study, 11 large nodules in 11 patients were completely ablated after single RFA, and 14 nodules with residual areas close to important structures or extending to clavicle in 13 patients underwent sequential RFA. In sequential RFA group, three nodules were close to trachea tightly, 10 ones extended to subclavian region, and one nodule was near to esophagus. One, 6, and 12 months after first RFA, patients in sequential RFA group, with residual nodules, received Ultrasound US and Contrast-Enhanced Ultrasound (CEUS) examinations to assess the therapeutic effect of the first RFA and blood supply to residual areas. These patients were arranged to receive sequential RFA treatment according to the reduction of ablation zone. Before RFA and CEUS examination, informed consents were singed by all cases, permitting to use their clinicopathological records for our research. The same group of interventional radiologists in our team carried out RFA procedures and recorded the treatment outcomes.

### Pretreatment assessment

We carefully recruited cases with histologically diagnosed benign thyroid nodules according to US-guided core needle biopsy (CNB) or at least two separate US-guided fine-needle aspiration biopsy (FNAB). Laboratory and clinical statuses for all cases were assessed by two interventional radiologists prior to therapy, whereas serum calcitonin degree was determined to rule out medullary carcinoma. Serum thyroid functions (free T3, free T4, and Thyroid Stimulating Hormone (TSH) levels) were checked before RFA and 1 day and 1 month after each RFA, respectively. US examinations were carried out for all patients prior to RFA and 1, 3, 6, and 12 months after RFA, respectively. For incompletely ablated nodules in sequential RFA group, 6 months and 12 months after the first RFA, CEUS would be implemented to assess therapeutic effects including blood supply to residual nodules and the reduction of ablation zone. The size, characteristics, and vascularity of every nodule were analyzed before and after each RFA. Three orthogonal diameters were determined between the outer margins of the nodule (23). The width and height of nodule were determined using axial image. Lengths were determined utilizing sagittal image (26).

### RFA procedure

Medsphere RF Generator S-500 with a 19-gauge, 7-cm length of shaft, 0.5-cm or 1-cm active tip internally cooled electrode (Medsphere, Shanghai, China) specifically designed for thyroid lesions was applied for RFA treatment, which made it easier to regulate and minimize normal tissue injury. RFA power was adjusted to 40 W for the first RFA using an impedance mode. For residual nodules that received sequential treatment, RF power was adjusted to 20 W.

Enrolled cases were fasted for one night and received operations in the morning. Prior to RFA, they lay down adopting a supine position with the neck mildly extending. Because discomfort may occur due to continuous injection of sterilized water into tracheoesophageal space, accompanied by large size of thyroid nodules, general anesthesia and tracheal intubation were applied to avoid any disturbances during RFA. Heart rate, blood pressure, respiration rate, and peripheral oxygenation were monitored during RFA. In cases with residual nodules, local anesthesia may be applied if residual nodule was less than 1.0 cm in diameter. US and CEUS were conducted before and immediately after each RFA (**Figures 1A, B, E, F**, **2A-C, B, F**, **3A, B, F**). Before RFA, appropriate route for electrode was determined to prevent serious hemorrhage, through carefully observing the location of vessels (anterior jugular vein). With real-time US guidance, percutaneous RFA was conducted on the basis of moving shot technique (**Figures 2E**, **3E**). Skin would not be incised to avoid unnecessary scars. At first, active electrode tip was placed into the deepest sites in nodules and went back to central and superficial regions. Through such procedures, nodules were destroyed under US surveillance.

### Multiplanar hydrodissection technique

Before RFA, sterile water was injected between thyroid and surrounding tissues such as skin, carotid artery, trachea, and esophagus, until thyroid tissues were fully isolated (around 2 mm between nodules and critical structures) (**Figures 1C, D**, **2C, D**). Then, RFA was conducted, and sterile water was continuously injected to keep thyroid tissues away from surrounding tissues during RFA. As ablation plane rose, sterile water needle was re-inserted into appropriate location. This multiplanar hydrodissection technique ensured the safety of RFA to treat large solid thyroid nodules at each plane.

Follows-up

We observed changes in internal vascularity and volumes for large thyroid nodules after each RFA treatment. CEUS examination was implemented for recruited cases 1 month after first RFA treatment (**Figure 1F**). US was conducted for all patients 1, 3, 6, and 12 months after each RFA. We assessed efficiency for each RFA through analyzing blood supply to nodules and examining volume reduction for treated thyroid nodules. Nodule volumes were determined through the following equation: volume = π*abc*/6, in which *a* referred to the largest diameter, and *b* and *c* represented other mutually perpendicular diameters (27). Volume alteration in treated nodules was assessed *via* US and CEUS detection. Volume reduction ratio = ([initial volume − final volume] ×100)/initial volume (28). Thyroid function and adverse events were also checked during the follow-up. Major and minor complications were determined on the basis of the Society of Interventional Radiology (29). Complications during and after RFA were handled adopting appropriate procedures (30). Before RFA, and 1 day and 1 month after each RFA, thyroid function indexes including free T3, free T4, and TSH levels were assessed to estimate the influence of RFA on thyroid function. Moreover, 6 months after each RFA, patients in both groups were appraised for their statuses in terms of obstructive and/or local pressure symptoms through comparing to baseline indexes (0 = none; 1 = insignificant; 2 = mild; 3 = remarkable).

### Statistical analysis

Data synthesis was carried out with SPSS 19.0 software. Results, such as alteration in largest diameters, volumes, volume reduction rates, and thyroid function, were compared utilizing Wilcoxon signed-rank test. Continuous data were represented by mean ± SD. P<0.05 represented significant threshold.

## Results

Twenty-four patients with 25 large solid thyroid nodules received single or sequential RFA treatment. Eleven nodules in 11 patients reached complete ablation after single RFA, whereas other 14 nodules in 13 patients underwent sequential RFA treatment. The initial volume of large nodules in single RFA group ranged from 25.0 to 87.3 ml, and such figures in sequential RFA group were between 26.1 and 203.4 ml. The initial volume of large nodules was smaller in single RFA group than in sequential RFA group. In sequential RFA group, the interval time between the first and second RFA treatments ranged from 6.4 to 20.1 months (**Table 2**). In sequential RFA group, three nodules were close to the trachea tightly, 10 nodules extended to subclavian region, and one nodule was near to the esophagus. The efficiency of single or sequential RFA is shown in **Tables 3**, **4**. In single RFA group, 1 month after RFA treatment, volume reduction ranged from 31.4% to 96.7% (mean ± SD, 78.6 ± 18.7%); and 6 months after RFA treatment, volume reduction ranged from 62.6% to 99.4% (mean ± SD, 93.6 ± 9.9%). In sequential RFA group, after the first RFA treatment, volume reduction ranged from 30.6% to 92.9% (mean ± SD, 67.4 ± 17.8%). One month after the second RFA treatment, volume reduction ranged from 52.3% to 95.5% (mean ± SD, 77.9 ± 11.4%), and 6 months after second RFA treatment, volume reduction ranged from 83.4% to 98.4% (mean ± SD, 94.8 ± 3.8%).

**Table 2:** Characteristics of enrolled patients with large benign solid thyroid nodules.

Variables	Single RFA group	Sequential RFA group
Patients		
Age(years), mean ± SD (range)	56.8 ± 12.6 years (43–85 years)	48.7 ± 14.2years (23–71 years)
Sex (male/female)	3/8	3/10
History of thyroid surgery	1	1
BTNs		
Location of BTNs		
Right lobe	7	5
Left lobe	3	8
Isthmus	1	1
Volume (ml), mean ± SD (range)	43.2 ± 19.3 ml (25.0–87.3 ml)	48.9 ± 44.4ml (26.1–203.4 ml)
Ablation time(s), mean ± SD (range)	664 ± 429.7 s (120–1450 s)	670.5 ± 319.6 s (300–1560 s)/276.4 ± 68.4 s (160–400 s)
Therapy interval time	/	10.0 ± 3.5 months (6.4-20.1 months)
Complications		
Pain	0	1
Vocal alterations	2	0
Massive bleeding	1	1
Extra-thyroidal hematoma	0	1

**Table 3:** The efficacy of singe RFA treatment.

Variable	Initial	1 month after single RFA	6 months after single RFA	P-value
Volume (ml)	43.20 ± 19.33 (25.02–87.34)	9.47 ± 10.12 (1.51–36.63) ^*^	2.29 ± 2.82 (0.31–10.84) ^#^	<0.0001^*^/< 0.05^#^
VRR (%)	/	78.6 ± 18.7 (31.4–96.7)	93.6 ± 9.9 (62.6–99.4)	<0.005

*Initial vs. 1 month after RFA; ^#^1 month after RFA vs. 6 month after RFA.

VRR, volume reduction rate; values are means ± standard deviations (range).

**Table 4:** The efficacy of sequential RFA treatment.

Variable	Initial	After first RFA	6 months after sequential RFA	P-value
Volume (ml)	48.89 ± 44.41 (26.09–203.37)	13.96 ± 11.35 (3.68–51.29) ^*^	2.01 ± 1.56 (0.98–7.20) ^#^	<0.005^*^/<0.005^#^
VRR (%)	/	67.4 ± 17.8 (30.6–92.9)	94.8 ± 3.8 (83.4–98.4)	<0.0001

*Initial vs. after first RFA; ^#^After first RFA vs. 6 months after sequential RFA.

VRR, volume reduction rate; values are means ± standard deviations (range).

In sequential RFA group, the interval time between two RFA treatments ranged from 6.4 to 20.1 months (mean ± SD, 10.0 ± 3.5 months). Therapy interval for one case was up to 20.1 months due to individual intentions, without marginal recurrence according to close ultrasound monitoring. Follow-up periods after the end of the last RFA were between 6.3 and 53.3 months (mean ± SD, 26.1 ± 15.6 months), with a median figure of 23.5 months. RFA power was adjusted to 40 W in all nodules in the first RFA treatment, and 20-W power was adopted for three nodules in sequential RFA. In single RFA group, ablation duration ranged from 120 to 1,450 s (mean ± SD, 664.0 ± 429.7 s). For sequential RFA group, ablation duration for the first RFA ranged from 300 to 1,560 s (mean ± SD, 670.5 ± 319.6 s), and in the second RFA treatment, the duration was between 160 and 400 s (mean ± SD, 276.4 ± 68.4 s).

**Figure 4:**
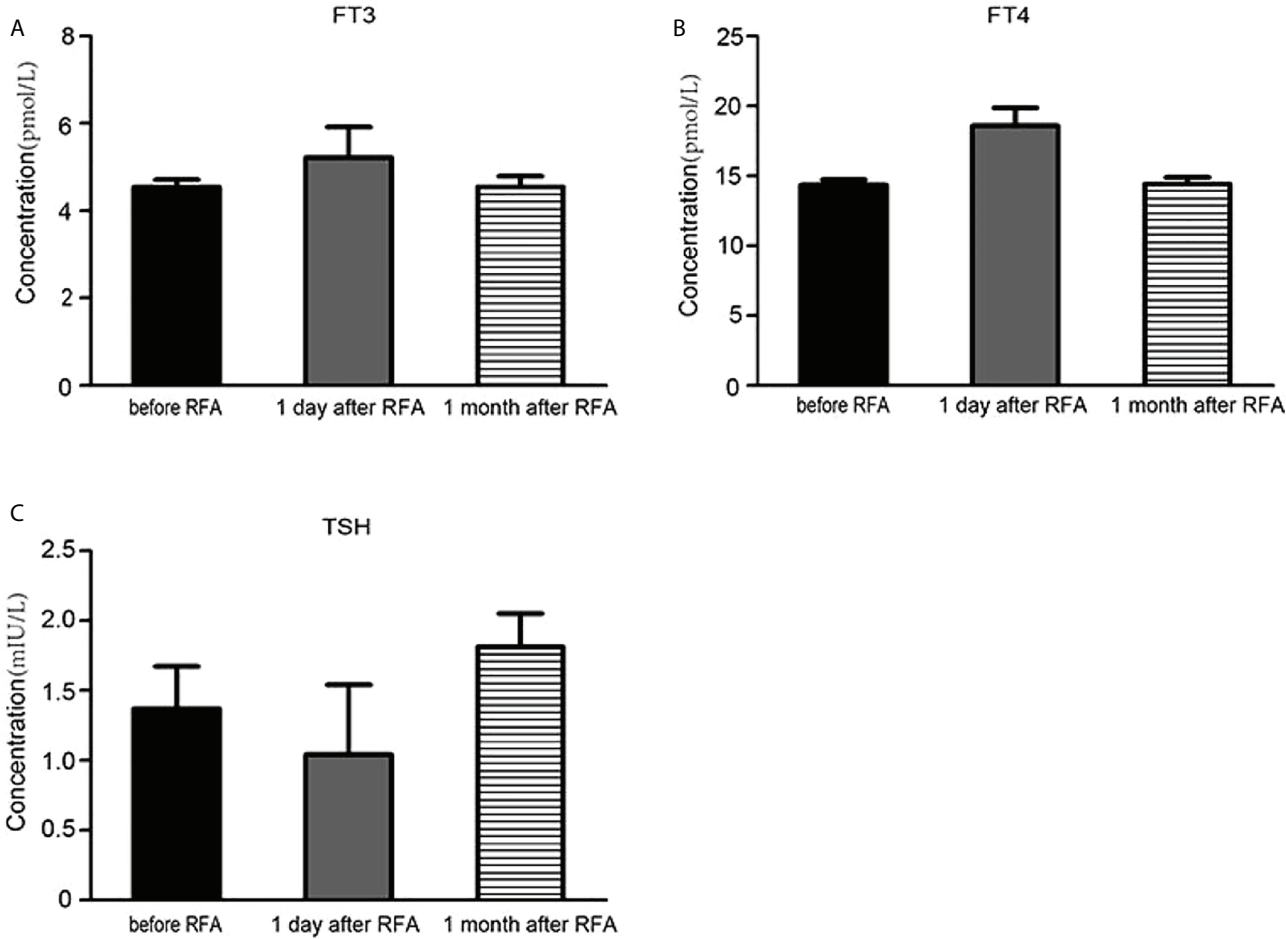
The influence of radiofrequency ablation on the thyroid function. **(A)** The serum FT3 concentration before radiofrequency ablation, 1 day and 1 month after radiofrequency ablation. **(B)** The serum FT4 concentration before radiofrequency ablation, 1 day and 1 month after radiofrequency ablation. **(C)** The serum TSH concentration before radiofrequency ablation, 1 day and 1 month after radiofrequency ablation.

Enrolled cases reached complete ablation, without relapse or enlargement 6 months following the last RFA. US test for each of enrolled cases at the last follow-up after RFA unveiled (1) distinctively reduced nodule volumes, (2) without internal vascularity at the margins of large solid nodules according to the Doppler US test, and (3) decreased echogenicity. The symptoms of airway compression improved significantly in all patients (**Table 5**). In addition, we also monitored changes in thyroid functions for cases holding large thyroid nodules before RFA, 1 day after RFA and 1 month after RFA. We found that the concentrations of FT3 and FT4 increased slightly 1 day after RFA and returned to normal levels 1 month after RFA, as shown in **Figures 4A, B**, when the concentration of TSH decreased slightly 1 day after RFA and also returned to normal degree 1 month after RFA, as shown in **Figure 4C**.

**Table 5:** Local pressure symptoms improvement scale.

Patient no.	Improvement scale from baseline
1	3
2	3
3	2
4	3
5	3
6	2
7	3
8	3
9	3
10	3
11	3
12	3
13	2
14	3
15	3
16	2
17	3
18	3
19	3
20	3
21	3
22	3
23	3
24	2

### Complications

One patient in sequential RFA group presented mild pains after sequential RFA and relieved within several hours. In single RFA group, two cases showed vocal alterations after RFA, but both recovered within 3 months following mecobalamine therapy. There were no voice changes in sequential RFA group. Inserting electrode into nodules caused massive bleeding in two cases due to damages on small vessels across puncture paths. The pressing of bleeding points successfully ended hemostasis, ensuring the success of RFA procedure. Following RFA, extra-thyroidal hematoma appeared in one patient and was settled within two weeks after proper management.

## Discussion

In clinic, follow-up observation is necessary after treatments for benign thyroid nodules. However, expanded volume of benign thyroid nodules not only affects the looks but also possibly compresses trachea, resulting in breathing problems. Therefore, timely and effective treatments are urgently needed for patients with large benign solid thyroid nodules. The definition for large thyroid nodules is not uniform at present. Some documents regard nodules with volumes under 10 ml as small ones and those more than this figure as large ones (28, 31, 32). Whereas some scholars claim that nodules with an initial volume more than 30.0 ml are large nodules (33). In our study, large solid thyroid nodule referred to those more than 25 ml.

RFA has been confirmed as an effective treatment for small- and medium-sized thyroid nodules, regardless of their constituents (23), but for large thyroid nodules, more caution should be taken considering their certain characteristics. First of all, the treatment with RFA of large thyroid nodules is more difficult and more dangerous. As we know, there are essential structures surrounding thyroid gland, like esophagus, trachea, recurrent laryngeal nerve, carotid artery, vagus nerve, and cervical sympathetic ganglion, and the edge of nodules could be very close to these configurations, especially for large thyroid nodules (34). Hence, it is difficult to treat large thyroid nodules safely and completely.

Hydrodissection technique has been widely used to minimize thermal damage through injecting sterile water between targeted nodules and bordering essential configurations during RFA (35). However, injected fluids may circulate fast along longitudinally neck muscle plane, so sterile water needs to be constantly supplied to maintain the separation of thyroid tissues from bordering ones during RFA. For large thyroid nodules, hydrodissection may be required at multiple planes during RFA. In this study, we adopted multiplanar hydrodissection technique through multiplane needle placement, and selective sequential RFA regimen was applied to ensure the safety and completeness of RFA in treating these large benign solid thyroid nodules. Through single RFA aided with multiplanar hydrodissection technique, complete ablation was achieved safely in 11 large nodules, but in other 14 large nodules located in fragile areas, sequential RFA was performed with full consideration of the safety, hoping to avoid cervical edema caused by hydrodissection technique, hypothyroidism caused by large ablation zone, and tracheal collapse caused by bilateral RFA treatment.

Second, large nodules should be ablated completely for the sake of preventing marginal recurrence. RFA can achieve outstanding outcomes in reducing volumes, alleviating compression symptoms and solving cosmetic issues, but according to previous reports, relapse rate ranged from 5.6% to 9% after RFA (28) and laser ablation (36), separately. Baek and colleagues (37) considered marginal re-expansion as a pivotal reason for relapse following RFA. Although some published articles have claimed predominant objective for RFA to treat benign thyroid nodules lies at decreasing compression symptoms, not full ablation (24), it is important to ablate nodules completely to eliminate the possibility of recurrence induced by margin regrowth of incompletely treated nodule (37–39). Well-treated nodules decrease by more than 90% over 1–2 years, whereas those not completely handed might grow within 1–2 years following ablation (28). Marginal re-expansion might represent one leading reason for relapse. Overall rate for regrowth (volume increase more than 50% in comparison to original status) has been up to 24.1% (32). Reported recurrence (increases more than 50% in comparison with earlier volumes according to ultrasonography) reached 57.4% (32). Accordingly, non-fully treated nodules might expand within 2 years following ablation, showing re-growth from their margins. Imperfect ablation for peripheral tissues might face relapse, and margins surrounding large thyroid nodules need to be wholly treated to minimize marginal recurrence. Furthermore, large nodules require multiple RFA sessions to achieve complete ablation.

Standards determining repeated RFA vary across different research studies including nodule residual during follow-up (23), volume reduction below 50% (23), and displaying partial compression symptoms (28). In our study, 1, 6, and 12 months after the first RFA, all cases with residual nodules underwent US and CEUS examination to determine reduction status for ablation region. The absorption of ablation zone makes subclavian parts of large nodules move up, creating greater feasibility and safety for sequential RFA.

**Figure 5:**
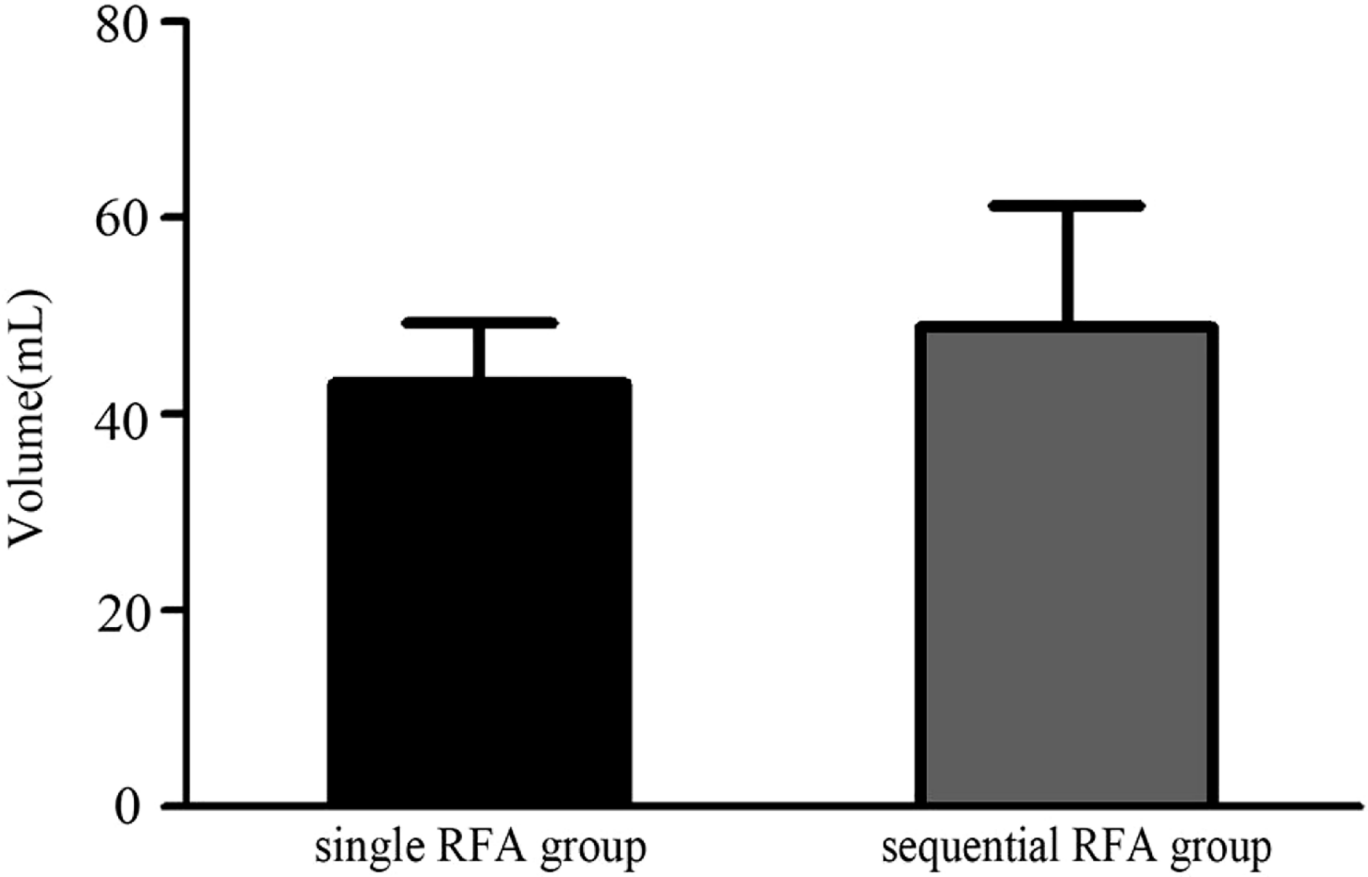
The difference of the volumes of the large solid thyroid nodules between single RFA group and sequential RFA group.

In our study, through single or sequential RFA aided with multiplanar hydrodissection technique, all large nodules reached complete ablation safely. Initial volume for large nodules in single RFA group was between 25.0 and 87.3 ml, and such figure in sequential RFA group was between 26.1 and 203.4 ml. Initial volume of large nodules was smaller in single RFA group than in sequential RFA group, as shown in **Figure 5**. All nodules in sequential RFA group were adjacent to important structures, when three of them were close to the trachea, 10 extended to subclavian region, and one nodule was near to the esophagus. Therefore, larger volume and risk location of such nodules may be main factors determining the frequency of RFA.

In our study, the complication rate of RFA was 25% (6/24). Owing to large size of thyroid nodules and heat concentration, thermal ablation may still cause thermal stimulation to peripheral nerves, despite the application of water isolation technique during the operation. We have explored some possible methods to reduce complications after RFA. Local hypothermia therapy was used to reduce nerve stimulation caused by postoperative heat. After the ablation, we applied ice bags at neck area for about 6 h to reduce local temperature. In addition, continuously injecting sterile water to isolate thyroid tissue from important surrounding tissues during RFA may lead to neck swelling and discomfort after RFA. To alleviate neck swelling and relieve local pain after surgery, we tried to apply traditional Chinese medicine mirabilite after local hypothermia therapy. The most serious complication in our study was vocal alterations that attacked two patients in single RFA group following RFA. However, in sequential RFA group, there was no such phenomenon, so sequential RFA may be one safer option for large and dangerously located nodules.

In our research, there were some shortages, such as lacking histological validation after treatment and possessing considerable similarities to other research studies focusing on non-surgery measures like ethanol ablation and laser ablation. To conquer such shortages, cases holding cytologically confirmed benign nodules should be enrolled—those who should be diagnosed on the basis of two separate US-guided FNAB or US-guided CNB and US imaging and exhibited no suspicious malignant characteristics. Another shortage lay in its retrospective design and small samples, only containing one single center. Sample sizes of two groups were relatively small, and no detailed treatment plan was developed for patients before ablation to satisfy the requirements of prospective design. Future prospective multicenter experiments are necessary to verify our findings.

## Conclusion

RFA is an efficient measure to treat large benign solid thyroid nodules, showing minimal invasion and reaching complete ablation to solve compression symptoms. Single or sequential RFA regimen might ablate large nodules with great safety and assurance. Single RFA assisted with multiplanar hydrodissection technique can achieve complete ablation in large thyroid nodules, whereas for larger nodules close to the trachea or extending to subclavian, sequential RFA treatment regimen might be preferred to avoid complications. Single or sequential RFA treatment can completely inactivate thyroid nodules with as little impact on thyroid function as possible and can solve cosmetic problems and compression symptoms safely.

## Data availability statement

The original contributions presented in the study are included in the article/supplementary material. Further inquiries can be directed to the corresponding authors.

## Author contributions

YL, YS, XT conceived and designed the experiments, analyzed the data, and wrote the paper. MD, YH, PL, BZ performed the experiments. All authors contributed to the article and approved the submitted version.

## Conflict of interest

The authors declare that the research was conducted in the absence of any commercial or financial relationships that could be construed as a potential conflict of interest.

## Publisher’s note

All claims expressed in this article are solely those of the authors and do not necessarily represent those of their affiliated organizations, or those of the publisher, the editors and the reviewers. Any product that may be evaluated in this article, or claim that may be made by its manufacturer, is not guaranteed or endorsed by the publisher.
